# CINS: Cell Interaction Network inference from Single cell expression data

**DOI:** 10.1371/journal.pcbi.1010468

**Published:** 2022-09-12

**Authors:** Ye Yuan, Carlos Cosme, Taylor Sterling Adams, Jonas Schupp, Koji Sakamoto, Nikos Xylourgidis, Matthew Ruffalo, Jiachen Li, Naftali Kaminski, Ziv Bar-Joseph

**Affiliations:** 1 Institute of Image Processing and Pattern Recognition, Shanghai Jiao Tong University, and Key Laboratory of System Control and Information Processing, Ministry of Education of China, Shanghai, China; 2 Machine Learning Department, School of Computer Science, Carnegie Mellon University, Pittsburgh, Pennsylvania, United States of America; 3 Section of Pulmonary, Critical Care and Sleep Medicine, Yale University School of Medicine, New Haven, Connecticut, United States of America; 4 Computational Biology Department, School of Computer Science, Carnegie Mellon University, Pittsburgh, Pennsylvania, United States of America; Allen Institute for Brain Science, UNITED STATES

## Abstract

Studies comparing single cell RNA-Seq (scRNA-Seq) data between conditions mainly focus on differences in the proportion of cell types or on differentially expressed genes. In many cases these differences are driven by changes in cell interactions which are challenging to infer without spatial information. To determine cell-cell interactions that differ between conditions we developed the Cell Interaction Network Inference (CINS) pipeline. CINS combines Bayesian network analysis with regression-based modeling to identify differential cell type interactions and the proteins that underlie them. We tested CINS on a disease case control and on an aging mouse dataset. In both cases CINS correctly identifies cell type interactions and the ligands involved in these interactions improving on prior methods suggested for cell interaction predictions. We performed additional mouse aging scRNA-Seq experiments which further support the interactions identified by CINS.

## Author summary

Single cell transcriptomics has emerged as a leading technology for studying the composition of organs and tissues in the human body, development and several other biological processes. More recent studies, including studies of various diseases (such as cancer), treatment—response studies and aging studies aim at comparing samples at the single cell level. To date, such analysis mainly focused on the differences in expression of genes in the different cell types. However, in addition to differences in expression such studies also provide information on the differences of cell type proportions between the conditions. To use such information for inferring cell interactions we developed a new computational framework termed CINS. CINS combines Bayesian network learning (which is used to infer cell type–cell type interactions) with constrained regression analysis (used to infer the specific proteins involved in such interactions). We applied CINS to a number of different case-control scRNA-Seq datasets including a lung disease and an aging study. By analyzing public data and profiling new scRNA-Seq data we show that CINS is able to correctly identify several proteins as playing a significant role in interactions controlling disease progression and aging, improving on prior methods suggested for this task.

This is a *PLOS Computational Biology* Methods paper.

## Introduction

The ability to profile the expression of genes at the single cell level has revolutionized gene expression studies. Single cell RNA-Seq (scRNA-Seq) studies resulted in insights related to the cell type composition of tissues [1,2], changes in cell type composition in various diseases and states [3], various differentiation pathways used within cells [4] and more. However, while scRNA-Seq provides valuable information about expression within cells, it is hard to use it to study interaction between cells. The main problem is that once cells are extracted it is very challenging to determine the spatial relationships among them [5].

A number of methods have been introduced recently to identify ligand receptor interactions in scRNA-Seq studies [6,7]. While these methods differ in the exact formulation and statical analysis, they all focus on finding correlations between ligands expressed in one cluster (or cell type) and receptors expressed in another. This works well for studies that are analyzing a single condition (for example expression in a specific tissue or at a specific time point) but does not fully utilize information in case-control studies single cell studies [8,9]. Unlike single condition studies, in addition to differences in expression case-control studies also provide information on differences in the *proportions* of different cell types between the conditions. Such information can be very useful in determining which cell types interact. When cell type proportions are correlated between two conditions (for example both high in one and low in the other) it may indicate that they are likely to interact [10,11]. As we show, this information greatly improves the ability to correctly infer cell-cell interactions from scRNA-Seq data.

In addition to methods that attempt to infer cell-cell interaction information from scRNA-Seq, a number of technologies have emerged for spatially profiling single cell expression data [12–15]. These technologies often combine Fluorescence in situ hybridization (FISH) with rapid sequencing to provide information on the spatial expression of thousands of genes at various resolutions [16,17]. A number of recent computational methods have been developed to allow for the study of signaling pathways involved in cell-cell interactions from this type of spatially-resolved expression data [18]. However, while spatial transcriptomics studies are promising there are several challenges involved in employing them to study intercellular interactions. First, current commercial spatial transcriptomics platforms do not profile cells at the single cell level. Most labs do not have access or ability to perform such studies at the single cell resolution. More importantly, spatial transcriptomics often requires the fixation of the samples which limits their usage and can negatively impact their ability to accurately profile molecular quantities [16]. Finally, spatial transcriptomics methods can scan only a small region of the tissue and so cannot be applied to large number of conditions and samples that are studied using scRNA-Seq.

Here we present a new method, the Cell Interaction Network Inference (CINS) pipeline, that infers cell type interactions in case control scRNA-Seq studies. CINS involves two major steps. First, it uses scRNA-Seq data from multiple samples of a similar condition (i.e. disease, age, etc.) to learn Bayesian networks which highlight the cell types whose distributions are co-varying under different conditions. Next, for the high scoring differential interactions identified in the Bayesian network analysis, CINS learns a regression model with ligand-target interaction matrix [6] that identifies the key ligands and targets that participate in the interactions between these cell types.

We tested CINS by applying it to both, disease and aging datasets. We show that CINS correctly identifies known interacting cell type pairs and ligands associated with these interactions and improves upon prior methods for inferring ligand-receptor interactions in scRNA-Seq data. We also discuss several novel predictions made by CINS. Finally, we show that a number of CINS predicted cell type interactions are supported by a new scRNA-Seq lung aging dataset we profiled.

## Results

### The Cell Interaction Network Inference (CINS) Pipeline

**Fig 1:**
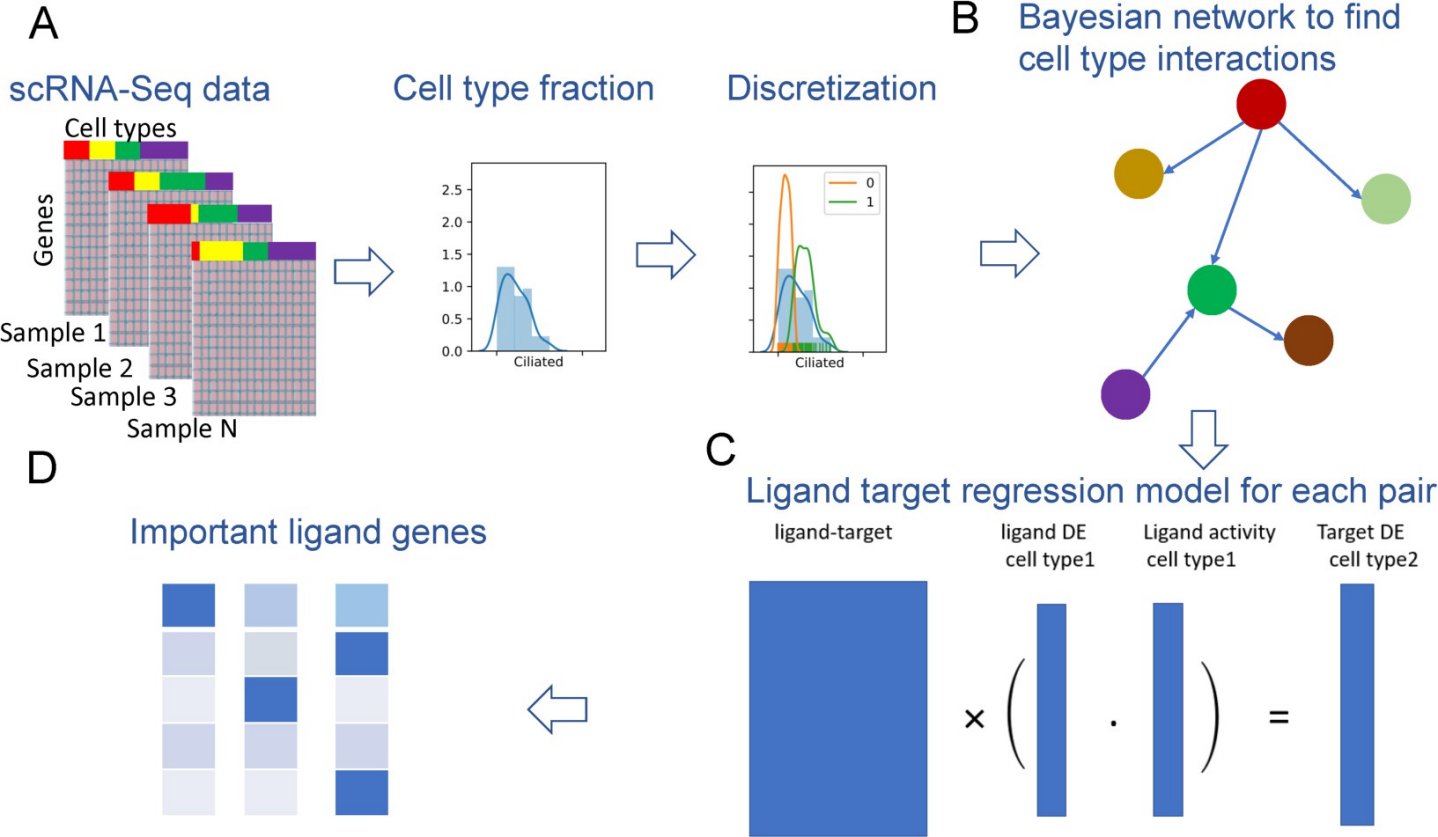
Overview of CINS. (A) Cell type annotation is used to extract cell type fractions in each sample. Next cell type fraction is discretized by learning Gaussian Mixture Model (GMM) for this type, respectively. (B) A Bayesian network (BN) is learned using the discretized cell abundance information. Bootstrapping is performed to identify high scoring differential interactions between cell types. (C) For pairs identified in the directed bootstrap BN analysis, a ligand-target regression (LTR) model is learned. In this model we use the expression change of ligands in the cell type with the outgoing edge to predict the expression of targets genes in the cell type with incoming edge. (D) Finally, LTR is used to select key ligands that underlie the cell-cell interactions identified in the BN. cell interaction.

We developed the Cell Interaction Network Inference (CINS) pipeline which uses single cell (sc) RNA-seq expression data to infer cell-cell interactions (**Fig 1**). Given repeated experiments of the same condition / system CINS uses annotated cell type information to construct a Bayesian network (BN) that models causal relationships between different cell types. For this, CINS first discretizes the proportion data for each cell type using a Gaussian Mixture Model (GMM) with only two components and then learns a BN that models the joint probability distribution of the cell type mixtures observed for each sample. High scoring differential causal relationships are determined based on bootstrapping. Next, for each of the high scoring differential pairs identified we infer the genes involved in the interactions by learning a ligand-target regression (LTR) model with ligand-target interaction database from NicheNet [6]. The LTR model aims to explain changes in target genes as a function of changes in their activating ligands allowing CINS to identify the most significant ligands that regulate the cell-cell interactions.

### Inferring cell type interactions using Bootstrapped Bayesian Network

We first applied CINS to simulated expression data. For this we generated cells using a fixed BN for cell-cell interactions and for each cell generated expression values based on their cell type. See **S4 Text** for details on the simulation settings and parameters. Results of our simulation analysis are presented in **S18** and **S19 Tables**. As can be seen, when not assuming dropout, CINS can correctly infer most of the underlying interactions (identifying 5 of 6 real interactions with accuracy of 83% and precision of 100%). While performance decreases when we increase noise, CINS is still able to accurately reconstruct the underlying BN even under relatively high noise levels (4 of 6 for 50% dropout with no false positive interactions).

We next applied CINS to a lung disease scRNA-Seq dataset [8]. The lung disease dataset contained scRNA-Seq data for 28 healthy (controls) and 32 Idiopathic Pulmonary Fibrosis (IPF) individuals. A total of 250,942 cells were profiled for these individuals. Cell type annotations were assigned based on the original study and we used the detailed assignments that provided information on 39 cell types.

**Fig 2:**
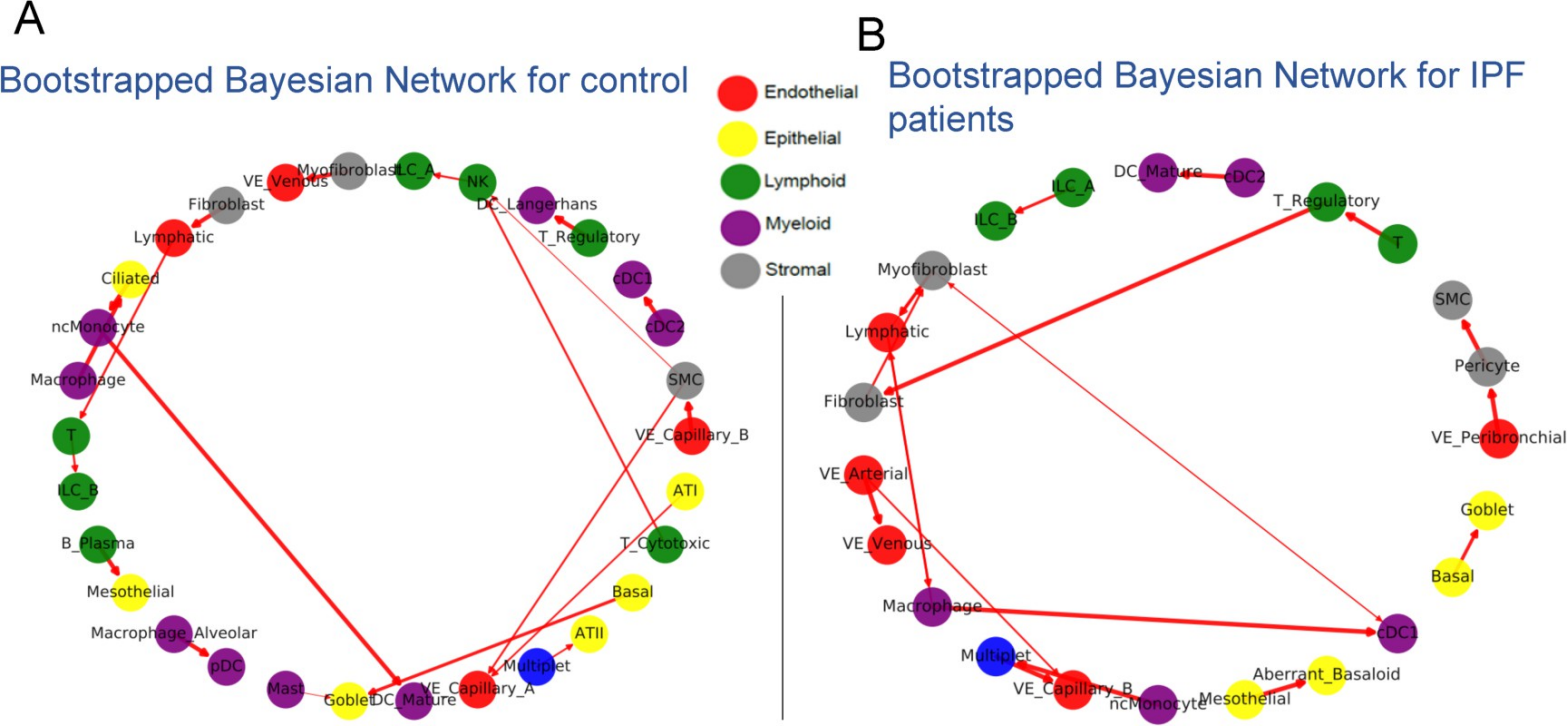
Bayesian Networks (BN) learned for lung cell types in healthy and IPF individual. (A) BN for controls (healthy individuals). (B) BN for IPF patients. Nodes represent specific cell types and are colored accordingly, edges represent directed interactions between the cell types. Edge width corresponds to its bootstrap score.

We used CINS to explore differential cell type interactions between IPF and control samples. For this, we constructed two different networks based on the cells profiled for each condition. We next performed bootstrap analysis to determine the score of each edge in each condition. Edges that appear in the majority of bootstrap iterations likely represent real relationships in the data rather than noise [19,20]. Resulting BNs for the two conditions are presented in **Fig 2A and 2B.** As the figures show, there are some edges that appear for both conditions. These include Basal to Goblet cell interactions, which agrees with the fact that club cell’s attachment sites are provided by Basal cell [21]. However, there are also many differences between edges selected for the two condition networks. **Table 1** summarized the top differences based on the signed difference in edge count in 100 bootstrap iterations for IPF and control (See **S1 Table** for differences for all detected edges). Several of the highest scoring edges are supported by prior work. For example, the edge from Treg to Fibroblast cell is supported by a previous study suggesting that Treg’s can negatively regulate fibroblast activity [22]. The edge between cDC2 and cDC1 is also supported by recent work showing that cDC2 and cDC1 are cross-talking with each other [23]. Several other top scoring edges are supported by the literature as referenced in **Table 1.** We next compared the interactions predicted by CINS to interactions predicted by CellPhoneDB, iTALK, and NicheNet (Methods), which are all popular methods for inferring ligand-receptor based cell interactions [6,7,24]. As can be seen, in **S2 Table**. unlike CINS which identified a diverse set of cell type interactions, almost all interactions predicted by CellPhoneDB involved Goblet cells (18 of the top 20). While there is some support for Goblet involvement in IPF [25] they only explain a small fraction (estimated to be less than 20%) of individuals with the disease and it is unlikely that they interact with almost all other cell types. Similarly, for NicheNet, almost all interactions predicted involved a single cell type, Pericyte cells. iTALK performed better, but it has only detected interactions between immune cells in the IPF lung dataset. While these are indeed of interest, the more interesting interactions are those between immune cells and fibroblast cells in the (injured) lung and none of these were identified by iTALK. In contrast, by looking at the overall distribution of cell types CINS was able to find a more general and, as we showed, accurate set of interactions between cell types that are likely relevant for the disease.

**Table 1:** Top differential cell type interactions identified by CINS for the IPF dataset. The IPF-Control column lists the difference in the number of times the edge between the two cells was identified in 100 bootstrap runs for each of the two datasets. Negative values indicate that it was identified more for the Control whereas positive numbers mean that the interaction is more prevalent in IPF. For all listed edges the interaction was only identified in one of the two datasets (score of 100 or -100).

cell_type1	cell_type2	IPF-Control	Reference
Macrophage	Ciliated	-100	There is strong interaction between ciliated cell and Macrophage in COVID-19 critical cases [44]
Fibroblast	Lymphatic	-100	Fibroblast produce extracellular matrix which is critical to lymph node microenvironment [45]
cDC2	DC_Mature	100	
cDC2	cDC1	-100	cDC2 and cDC1 are cross-talking with each other [23]
Macrophage	cDC1	100	
Mesothelial	Aberrant_Basaloid	100	
Macrophage_Alveolar	pDC	-100	Macrophage_Alveolar (AM) and pDC are involved in antiviral immune, and pDC will be activated if the AM defense line is broken [46]
Myofibroblast	VE_Venous	-100	Injury lets endothelial cells transform to myofibroblast [47]
Ciliated	ncMonocyte	-100	Ciliated cells may contribute to monocyte inflow in COVID-19 [44]
Multiplet	VE_Capillary_B	100	
B_Plasma	Mesothelial	-100	Excess plasma cells are found with mesothelial cells on effusion cytology smear [48]
VE_Capillary_B	SMC	-100	
Pericyte	SMC	100	Brain pericytes and vascular SMC comprise mural cells which is important to support blood vessels [49]
ncMonocyte	Multiplet	100	
ncMonocyte	DC_Mature	-100	
T_Regulatory	Fibroblast	100	Treg cell regulates fibroblast in lung [22]
VE_Arterial	VE_Venous	100	
T	T_Regulatory	100	
VE_Peribronchial	Pericyte	100	One pericyte can communicate with more than one endothelial cells [50]
T_Regulatory	DC_Langerhans	-100	

### Inferring ligand-target interactions for high scoring differential cell type pairs

While the BNs discussed above identify pairs of cell types that likely interact in disease, the network does not show which genes and protein products participate in the interactions. To infer such gene-gene interactions across cells we developed a ligand-target regression (LTR) model. For cell type pairs identified in the BNs our LTR model uses a set of ligands in the first cell type to predict the expression values of their known targets in the second cell type. The LTR model uses the LASSO algorithm which enables the identification of a small set of key ligands predicted to participate in the interaction observed in the BN. We trained the model using a five-fold cross validation strategy. See **Methods** for details.

The LTR method was applied to all high scoring differential pairs identified by the BN. **S3 Table** presents top scoring ligands for several cell type pairs. **S4 Table** presents top scoring ligands for one cell type pair (Fibroblast -> Lymphatic cell). Several of the top LTR ligands are known to play an important role in the activated cell (Lymphatic cell). For example, the highest scoring ligand identified by LTR is “FGF2” which was identified as a critical gene for lymphangiogenesis [26]. Another highly ranked ligand, “TGFB1”, can also accelerate lymphatic regeneration in wound repair [27]. **S5 Table** presents top ranked ligands for another pair (Treg cell -> Fibroblast), several of which have also been shown to participate in the interaction between these cell types. For example, fibroblast express IL13 receptor and may behave as an inflammatory cell if stimulated by IL-13 [28], and TGFB1-3 (including TGFB1 and TGFB2 in the table) are all involved in promoting collagen production in fibroblasts [29].

### Identified ligands are primarily involved in cell-cell interactions

To test if the predicted ligands are indeed impacting cell type-cell type interactions or mainly represent autocrine relationships we compared the activity of top predicted ligands within and between cell types. For this, we compared the performance of the LTR method for top edges to the performance of a similar method that only uses information from a single cell type. Specifically, if the BN predicted a high scoring differential interaction between cell types A -> B, we first trained LTR using the ligands of A and the targets of B (as we did above) and compared the performance to a LTR model which uses the ligands expressed in B to predict targets in B (autocrine model).

**Fig 3:**
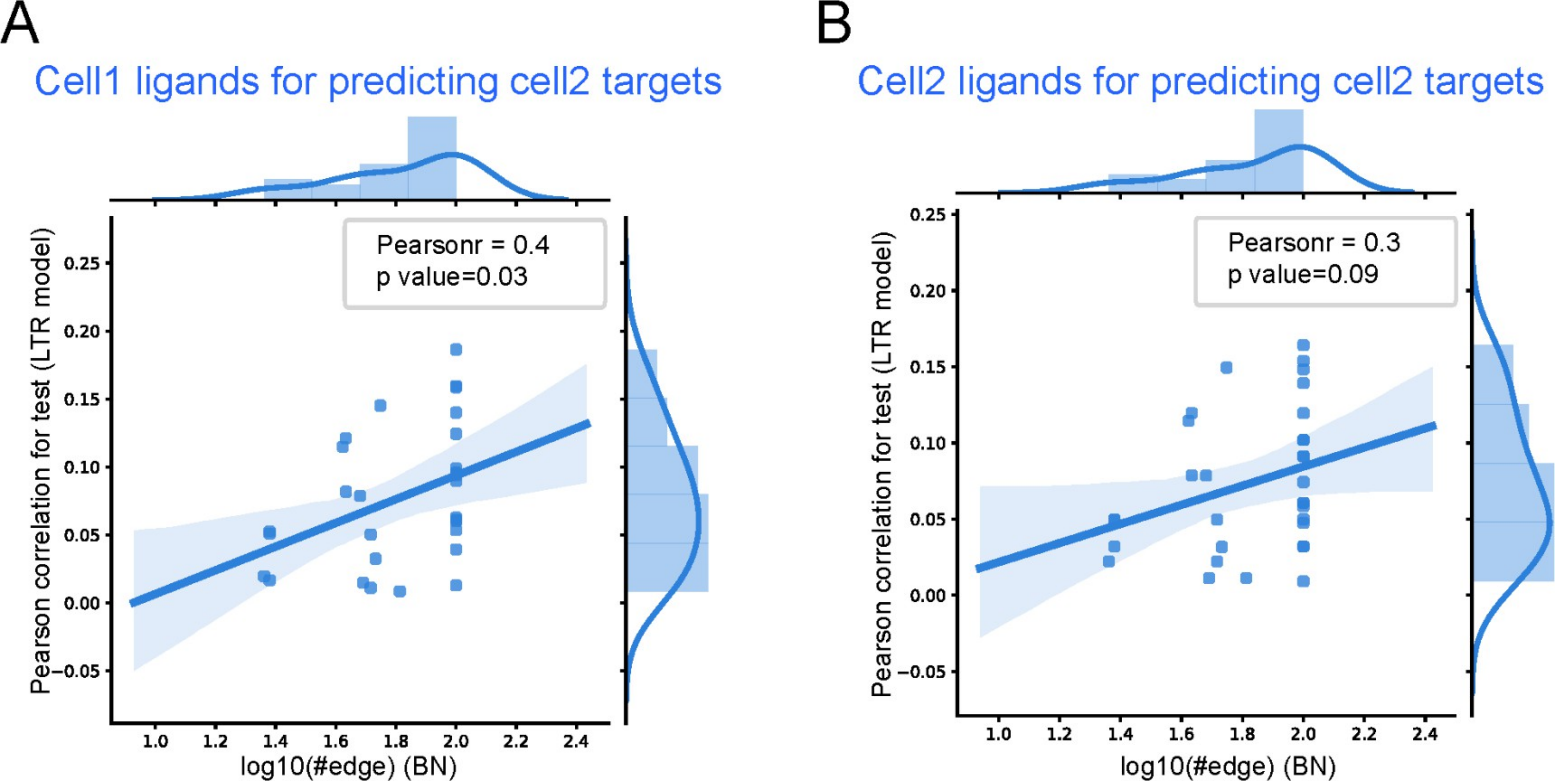
Interactions learned by the BN are more significant than interactions between cells of the same type. Comparison between the ability of the LTR model to predict target expression change when learning the model using cell pairs identified by the BN (A) and the same cell type (B). The x axis represents the bootstrapped edge count (score) of the interaction in the BN for a cell type pair, and the y axis represents the LTR model performance (higher is better) for the same cell pair.

Results for the high scoring differential edges in the IPF and control datasets is presented in **Fig 3A and 3B.** presents the results for the same pairs (so x axis is fixed based on the BN score) but with the LTR trained using only the ligands of the second cell type. As can be seen, when using the ligand of the predicted interacting cell type LTR obtained a higher average correlation with a p-value of 0.034 (using the scipy function in Python for computing Pearson correlation p-values). In contrast, when using the same cell type for both ligands and targets the Pearson correlation is lower (**Fig 3B)**. We also evaluated the performance of the LTR method on the predicted cell type interactions by comparing the results we obtained with the real ligand-target interaction matrix to results obtained using a random ligand-target interaction matrix. We found that for most of the random assignments the resulting LASSO models contained only a Bias term with all coefficients set to 0 (**S3 Fig**). This indicates that expression of the ligands did not provide any useful information about the expression of the targets when using the random interaction matrix.

### Application to a scRNA-Seq dataset on lung aging

**Fig 4:**
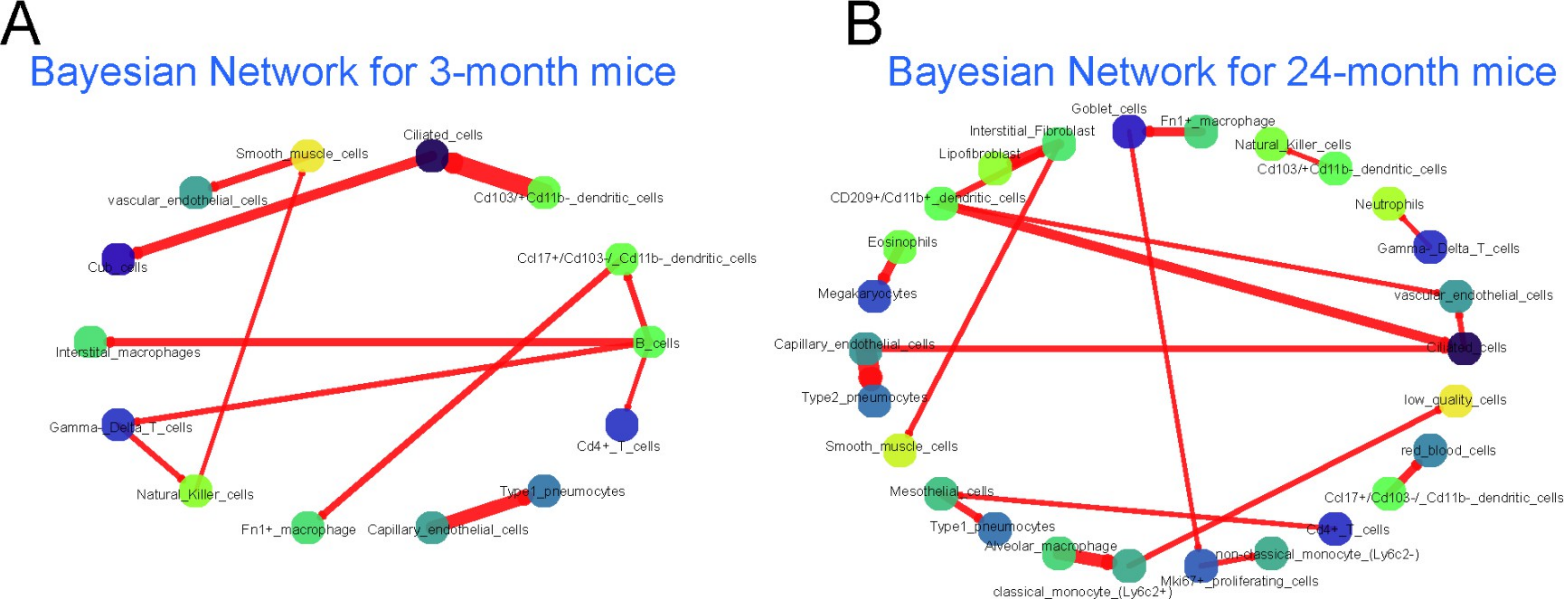
Aging Bayesian Networks. (A) BN for young mice. B) BN for adult mice. Nodes and edges notations and colorings are similar to those used in **Fig 2**.

We next applied CINS to another, smaller, scRNA-Seq dataset which studied lung aging in mice [9]. The dataset profiled lung cells in 15 mice, 8 young (three-month, 3M) and 7 old (24-month, 24M). The 14,813 cells profiled in this study were assigned to one of 34 cell types in the original paper. We again learned 100 bootstrapped BNs for the two conditions (young and old) and compared the resulting networks. We found 11 edges to be differentially present between the two conditions when using an edge threshold count of 20 (**Fig 4** and **S6 Table**). These included an edge between Capillary-endothelial-cell and Type 1-pneumocyte cells which are known to jointly form thin air-blood barriers used for gas exchange [30]. Another pair was Ciliated and Club cells, of which the ratio is reported to alert significantly between young and old mouse lung [9]. We next performed LTR analysis on the high scoring differential edges. The top ranked ligand in Ciliated cells, TNF is known to regulate CC16 gene production, which plays a role in immunomodulatory activity in Club cells [31]. Apoe, a ligand identified for the macrophage to goblet edge, is produced by macrophages to negatively modulate goblet cell hyperplasia [32].

**Fig 5:**
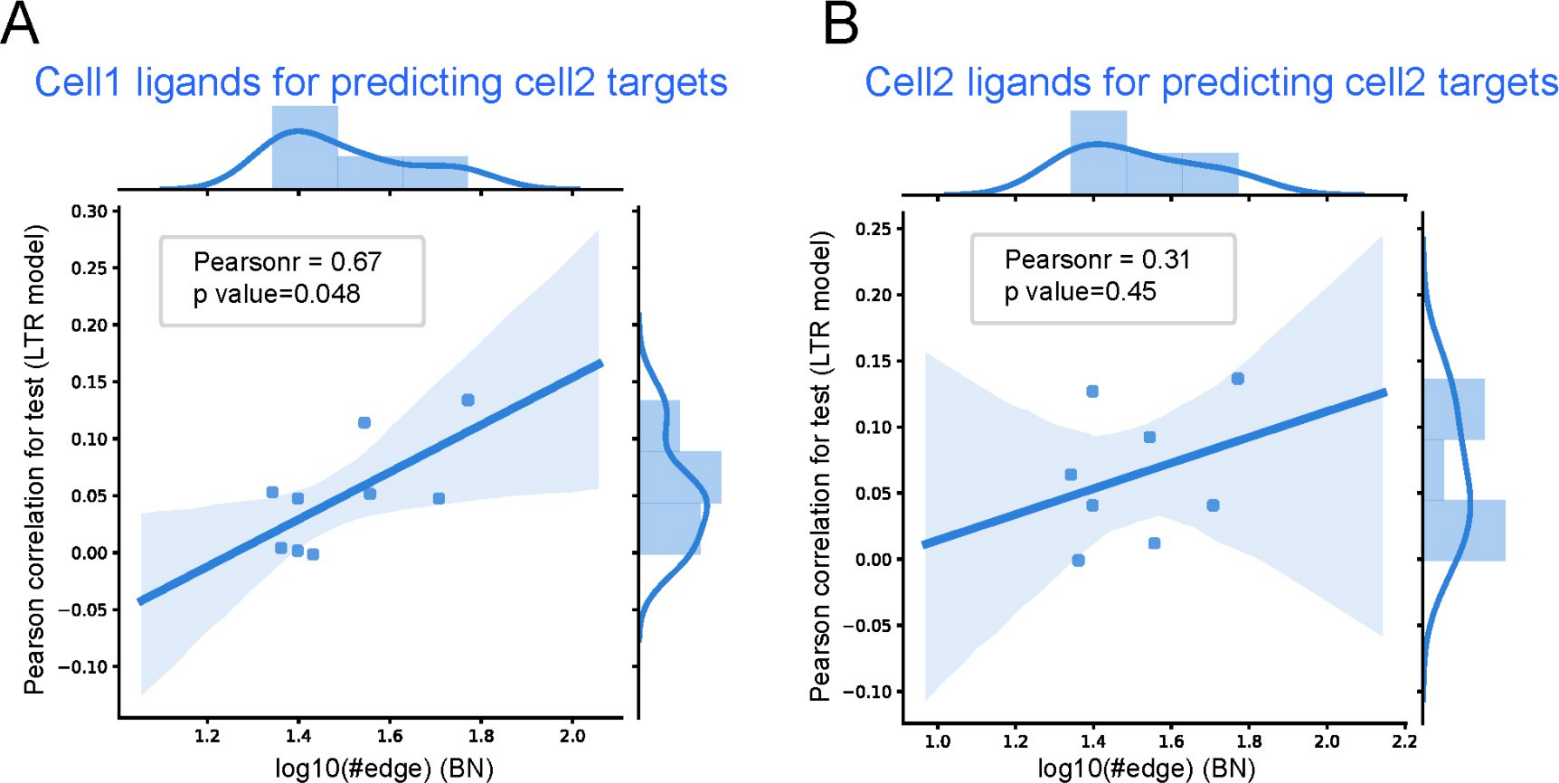
LTR comparison for the aging data. Comparison between the ability of the LTR model to predict target expression change when learning the model using cell pairs identified by the BN (A) and the same cell type (B).

As we did for the IPF study we compared the performance of the LTR method using ligands from the BN identified edges (A -> B) and ligands from the same cell type (B) to predict target expression for genes in B. We observed a Pearson correlation of 0.67 when using the ligands from the BN identified edges (A->B) vs. Pearson correlation of 0.31 when using the ligands from B (**Fig 5**). And it is noticed that when randomizing the interactions the LTR method again failed to identify any significant correlation between predicted and real expression for the targets (**S3 Fig**).

### Computational validation of high scoring differential edges using a second aging mouse lung dataset

To test the predictions of the aging BN and to validate them using an independent cohort we next performed additional scRNA-Seq experiments on young and old mice to generate a pilot scRNA-Seq dataset on lung aging. For this, we profiled four young and four old mice of the *Fendrr-floxed* genotype recently generated in the Kaminski laboratory. We obtained 71,562 cells that were clustered, annotated, and assigned to 20 cell types that overlapped with the cell types assigned by Angelidis I et al. [9]. The problem with both aging datasets is their small size 15 and 8 compared to 60 in IPF dataset). We could not obtain significant results using the 8 dataset aging data given its small size. Thus, we could not use it as a standalone dataset to validate the results of the larger (15 samples) datasets. Instead, we looked at the impact of combining the two. We next used the combined data (from [30] and from our new experiments) to learn a joint BN. Several of the predicted interactions were further supported by our new data. Specifically, we found 19 cell type pairs for which the addition of our new data enhanced both the presence of the edge and the direction predicted when performing the bootstrap analysis. **S8 Table** presents the top 10 enhanced pairs based on the overall bootstrap score (See **S11 Table** for all enhanced pairs). For example, the interaction between Neutrophils and Gamma Delta T cell is enhanced from edge count of 40 to 61 and was reported by recent studies that neutrophils can suppress Gamma Delta T cell’s activation involved in the resolution of inflammation [33]. And the interaction between B Cell and CD4+ T Cell is enhanced from -16 to -19 (being negative means that old lung has less), and is supported by other studies that B cell will activate CD4 T cells in human cutaneous leishmaniasis infection led by Viannia [34]. In addition, we also found that T-cell-B-cell interactions were calculated to occur less often in older samples, which further validates the comparison between old and young mice [35].

**Fig 6:**
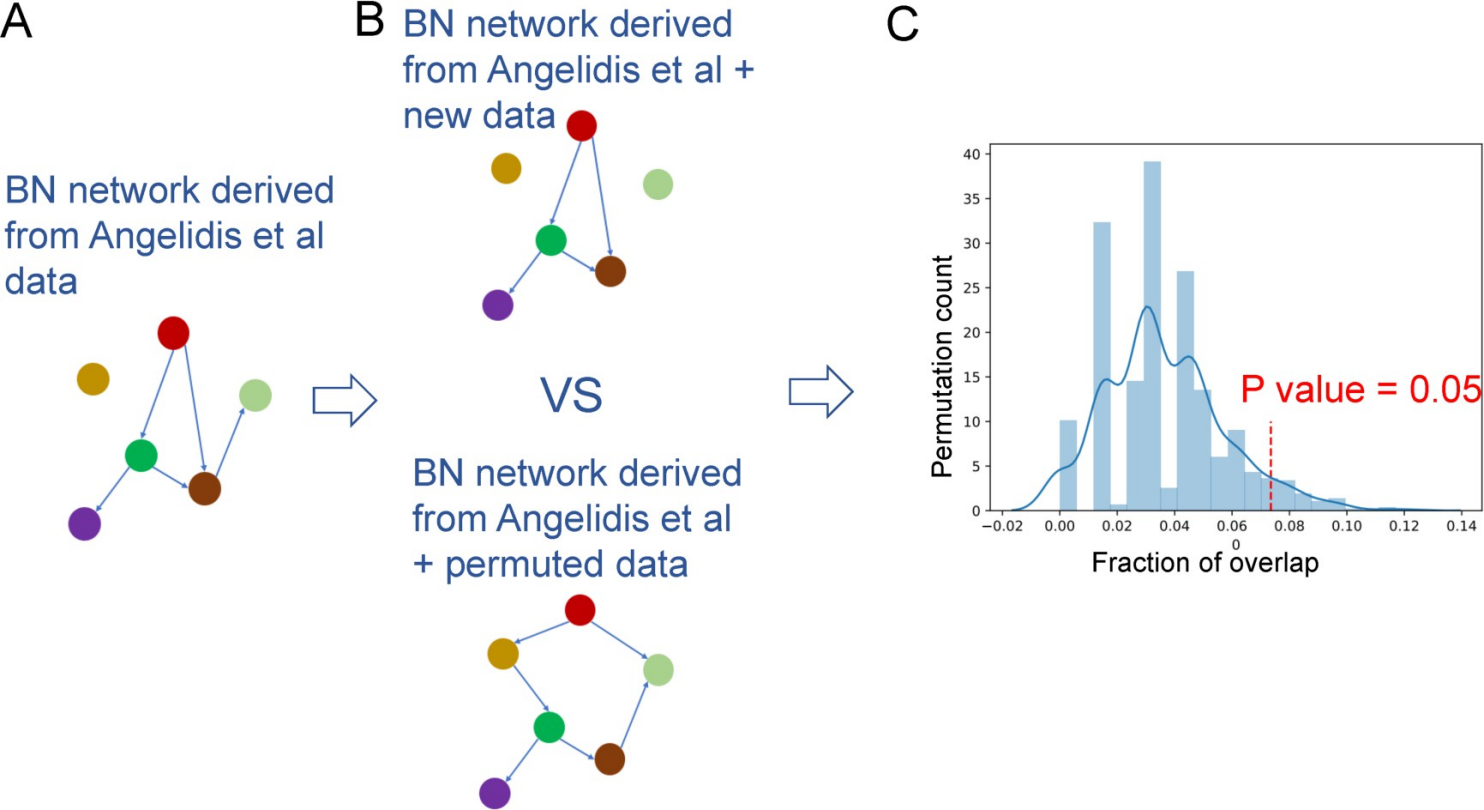
Permutation analysis highlights the agreement between the two aging networks. (A) Leftmost–learning using the Angelidis (15 samples) dataset. (B) Top–Learning combined networks using both Angelidis and real new data. Bottom–Learning combined networks using both Angelidis and permutation of cell type fractions in the new data. (C) Overlap in bootstrapped edges between the original and combined model when using the real data (red dashed line) and the permutation data (blue distribution).

We next focused on the top five predicted interactions in **S8 Table** (all with an absolute enhanced bootstrap score larger than 15). Permutation analysis indicates that identifying such a large number of edges supported by both studies is significant (p-value = 0.05, **Methods** and **Fig 6,** and see **S13 Table** for result of other threshold values**)**. Specifically, we permutated the cell type fraction of the aging dataset with 8 samples, and then did the BN analysis for 1,000 times. We next calculated the fraction of enhanced pairs with certain edge threshold over the whole pairs reported. We applied LTR to the cell type pairs in **S8 Table** to find important ligand genes. **S9 Table** presents the top predicted ligand genes. Several of these (red font) are supported by prior studies on the interaction between these cell types. Comparisons to CellPhoneDB, iTALK and NicheNet indicated that, similar to what we observed for the IPF data, the predicted interactions are very different compared to CINS (**S4 Fig**). In addition, unlike CINS for which the overlap between the pairs identified with and without the new datasets were significant, for CellPhoneDB we did not observe significant overlap between predicted interaction pairs (**S13 Table)**.

## Discussion

To enable the study of cell type–cell type interactions using scRNA-Seq data we developed a method termed Cell Interaction Network Inference (CINS). CINS first learns a Bayesian network between cell types (BN) using repeated samples. High scoring differential cell type pairs identified by the BN are further studied to infer the ligands that regulate these interactions. CINS is implemented in python and R and can be downloaded from https://github.com/xiaoyeye/CINS.

While CINS can be applied to any dataset with multiple samples, it is most appropriate for datasets containing case and control or multiple conditions. For such datasets CINS can infer not only the high scoring differential interactions within a condition but also those interactions that differ between the condition and that may partially explain the differences between the conditions studied. The main difference between CINS and all prior methods is the unique ability of CINS to take advantage of case–control studies to infer cell type interactions. Prior methods mainly focus on ligand gene expression information of each of these datasets separately and do not use the changes in fraction between the case and controls to infer such interactions, while CINS can make use of cell proportion as additional information and the two can prove each other mutually, further confirming the findings. The discretization of cell proportion can fit the data very well and makes it easier for BN to learn the correct structure of the network which is the major focus of CINS. As we show, by using the case control setting the interactions we can obtain are indeed more accurate and more diverse than these prior methods.

We first applied CINS to simulated data and demonstrated its accuracy on simulated NB networks. We next applied CINS to study a case and control dataset profiling lung expression from IPF patients and controls. CINS identified several differences between the interactions observed for IPF patients and for healthy individuals. These include the interaction from Treg to Fibroblast cells which is supported by a recent study that found Treg can negatively regulate fibroblast activity [22], and the edge between cDC2 and cDC1 is also supported by recent work showing that cDC2 and cDC1 are cross-talking with each other [23].

For many of the identified high scoring differential interactions CINS was also able to identify key ligands involved in the interactions. For example, “FGF2” which was identified as a critical gene for lymphangiogenesis [26], and one more highly ranked ligand, “TGFB1”, can also accelerate lymphatic regeneration in wound repair [27].

We next applied CINS to a lung scRNA-Seq aging dataset and identified a number of high scoring differential pairs that differ between young and old mice. To validate predicted interactions we performed additional experiments in which we profiled scRNA-Seq expression in 4 additional young and old mice and then used the combined dataset to learn a joint network. As we showed, the network we learned identified a significant number of interactions that are supported by both datasets. These include the interactions between Neutrophils and Gamma Delta T cell [33], and between B Cell and CD4+ T Cell [34,35] which are both supported by previous studies. CINS was again able to identify key ligands involved in these interactions, TNF, identified as the top ligand in the interaction between neutrophils and Gamma Delta T cells was previously identified as expressed in neutrophils [36] and as a regulator of immune cells Gamma Delta T cells [37], and TNFSF18 identified in interactions between CD4+ T cells and Vascular Endothelial Cells, was also previously reported to mediate the interactions between immune cells and endothelial cells [38].

While CINS can be successfully applied to several scRNA-Seq studies, there are still many places where it can be improved. First, it can only be applied if multiple samples are profiled since the BN part requires several repeated samples to compute relationships between cells. In addition, because BNs do not allow self edges, interactions between cells of the same type cannot be identified by CINS. Thirdly, since it uses a bootstrap approach to infer edge score it can miss important interactions if not enough samples and / or cells are available. Finally, CINS indeed requires, and relies on, prior cell type annotations. Since most case control datasets are from the same experiment, differences between cell type assignments attributed to individual preference should be minimal. If such information is not provided users would need to annotate their cells using one of the cell atlas annotation servers (for example, scQuery [1,2]) and then apply CINS.

CINS is one of the first methods to enable the inference of cell type interactions in scRNA-Seq data from *repeated* samples. Given the growing popularity of this method, and its increased use in clinical studies which are currently less amenable to spatial transcriptomics techniques we believe that CINS provides a solution to an important problem that is not currently addressed.

## Materials and methods

We developed a pipeline for modeling interactions between cells of different types from scRNA-Seq data. Our method first identifies cell types that are likely interacting and then tries to provide a mechanistic model to explain how such interactions are manifested at the molecular level.

### Datasets

We tested CINS using three scRNA-Seq datasets. The first compared gene expression in lungs of healthy and Idiopathic Pulmonary Fibrosis (IPF) with accession number of GSE136831 [8]. This dataset contained 28 controls and 32 IPF patients with a total of 243,472 cells and the expression levels for 45,947 genes in each cell. We used the original annotations and included in the model all 39 cell types with at least 100 cells. The second dataset studied lung aging in mice with accession number of GSE124872 [9]. This dataset contained 8 three-month-old mice and 7 24-month-old mice for which a total of 14,813 cells were profiled. For each cell the expression levels of 21,969 genes were provided. Each cell was assigned by the authors to one of 34 cell types. The third dataset was a new dataset in which we profiled single cell expression in four young (25 weeks) and four old (2x 103 weeks; 2x 120 weeks) *Fendrr-floxed* mouse lungs. This dataset contained a total of 71,562 cells with expression values for 45,947 genes. These cells were originally assigned to 37 cell types based on the expression of canonical cell type markers. To combine the two aging datasets we did the following. We first normalized the gene expression data using the same method for both datasets. Next, we manually assigned a common set of cell types to both datasets so all cell type match between the two. Specifically, we identified a joint subset of 20 cell types identified by both and only used cells assigned to these cell types in our combined BN analysis (see **S10 Table** for cell type information details). Information about ligands and their targets were obtained from a recent paper [6] which provided targets for 688 ligands.

### Single-cell sequencing of *Fendrr-floxed* mice

Animal procedures had been approved by the Institutional Animal Care and Use Committee (IACUC). We created a floxed allele of *Fendrr* via two-guide, two-oligo CRISPR/Cas mediated cleavage and recombination essentially as described in Yang et al. [39]. A generated mouse which had the expected conditional allele was bred with C57BL/6J mice to establish the colony and to sort the floxed allele from any other possible mutant alleles. Three female and five male mice in two age groups (young: 23 weeks, old: ranging from 103 to 120 weeks; four mice per group) were euthanized, and lungs were harvested and minced in small pieces with a scalpel. Lung pieces were dissociated using the enzyme Liberase TL (Roche).

Single RNA molecules of single cells were barcoded using the 10× chromium single-cell technology according to the manufacturer’s instructions (Single Cell 3′ Reagent Kits v2, 10× Genomics, USA). Barcodes were used to assign reads to cells and quality control was performed to remove low quality cells (S2 Text). Generated sequencing data is available at GEO accession number GSE165638. A modified version of the standard Seurat pipeline was employed to normalize, cluster and annotate the raw counts single-cell expression data for downstream analysis [40]. Briefly, the percent of mitochondrially-expressed genes was calculated for each individual cellbarcode using the *PercentageFeatureSet* function. Next, unique molecular identifier (UMI) counts were log normalized with a scale factor of 10,000 UMIs per cell and then natural log transformed using a pseudocount of one. Following log normalization, the top 3500 variable genes within the dataset were determined using Seurat’s implementation of the *FindVariableFeatures* function with the “vst” parameter. Next, the gene-level scaling of the data was performed using the *ScaleData* function. Each feature was centered to have a mean of zero and scaled by the standard deviation of each feature. The percent of mitochondrially-expressed genes captured within each cell were regressed out during scaling by using the “vars.to.regress” parameter. To reduce the dimensionality of the dataset and to identify genes contributing the most variability to the underlying manifold of the dataset, Principal Component Analysis (PCA) was performed using the scaled data and the 3500 variable genes calculated determined for the dataset. Following exploration of the PCs (S3 Text), the first 75 PCs were selected for clustering and following Uniform Manifold Approximation and Projection (UMAP), a dimensionality reduction method. The quality of subject and age representation within each cluster was assessed prior to cell type annotation to note any subject- or age-specific biases.

### Cell type assignment of *Fendrr-floxed* mice

To assign a specific cellular identity to each cluster, differentially expressed markers were determined and assessed within the context of canonical marker genes. Briefly, a differential gene expression test using Wilcoxon Rank Sum test was performed that compared the gene expression within a specific cluster to expression within all cells outside of that cluster. The resulting list of cluster-specific marker genes was assessed and cell types were ascribed based on expression of canonical marker genes. Clusters displaying canonical markers for multiple cell types were flagged as multiplets and were omitted from downstream analysis.

### Cell type quantification and discretization

We use the cell type annotation information provided by each study. To use Bayesian network to learn relationships between cell type we first discretize the proportion of each cell type in each sample. Discretization is cell type specific (i.e. different cell type will be assigned different values for the same proportion quantity) and is learned using an unsupervised method based on Gaussian Mixture Model (GMM) with two components. Specifically, let $\left[x_{1}^{i},x_{2}^{i},\cdots x_{n}^{i}\cdots ,x_{N}^{i}\right]$ be the fraction (percentage) of the *ith* cell type in the N samples. We learn a two components GMM for these values and then assign each value to the class with the higher likelihood for this value. The target function of the GMM aims to maximize the log likelihood:

$$l^{i}(\pi^{i},\mu^{i},\Sigma^{i})=\sum_{n=1}^{N}log(\sum_{k=0}^{1}\pi_{k}^{i}N(x_{n}^{i},\mu_{k}^{i},\sigma_{k}^{i}))$$ (1)

Where N represents gaussian distribution and ($\pi_{k}^{i},\mu_{k}^{i},\;\sigma_{k}^{i}$) represent proportion, mean and standard deviation parameters for the *kth* component of the *ith* cell type.

Following convergence, each proportion value $x_{n}^{i}$ is assigned to one of the two classes. We assign labels to the two classes such that the component with lower mean parameter is assigned a value of 0 and the second is assigned a value of 1. Specifically, the cell type specific cutoff is determined by the GMM model, which can automatically find the cutoff based on the clustering it learns. Next, samples assigned to the cluster with the higher mean are set to 1, and those assigned to the lower mean cluster are set to 0. This leads to a learned cell type specific cutoff such that all samples with a value less than that cutoff are assigned to 0 and all those above are assigned to 1. However, the number of 0’s and 1’s is not pre-determined and may be highly skewed in either direction based on the distribution of the fractions. See **S1 Fig** for examples of assignments. To learn GMMs we used the Python package “sklearn” with a maximum iteration number of 500 and a convergence threshold of 10**-4.

### Learning a cell type Bayesian network

We use the discretized cell type values to learn a cell type Bayesian network. Bayesian network is a probabilistic graphical model that uses directed acyclic graph to represent joint probability distributions. The absence of an edge can indicate independence and / or conditional independence. Bayesian networks are parameterized as <*G*, *P*> where G = <*V*, *E*> is a directed acyclic graph with *V* as variables and E as directed edges, and P is the global joint distribution for all nodes *V*. Given the graph structure this probability can be decomposed into local distribution for each node, *V*_*q*_, conditioned on its parent nodes as follows:

$$P(V|\Theta ,G)=P(V_{1},V_{2},\ldots .,V_{Q}|\Theta ,G)=\prod_{q=1}^{Q}P(V_{q}|Pa(V_{q}|\Theta_{q}))$$ (2)

Where *Pa*(*V*_*q*_|Θ_*q*_) is parent node set of *V*_*q*_ according to *G*.

To learn a Bayesian network using the discretized cell type proportion data, we iterate between network learning and parameter estimation. We initialize the network using the *Hiton Parents and Children* strategy which is based on marginal association among variables [41]. Next we iterate a search strategy, that uses penalized Hill-Climbing to add, flip or remove edges based on the Bayesian Information Criterion (BIC) score when using dataset *D*, where each sample in *D* contains values for all the variables of *V*:

$$BIC=logP(D|\Theta ,G)-\frac{1}{2}Dim(G)logN$$ (3)

where *N* is the number of samples and *Dim*(*G*) is the number of parameters in the model. For this, we used the “rsmax2” function from the R library “bnlearn”, which implements the iterative Penalized Maximization algorithm to construct a Bayesian network.

To obtain confidence values for edges (predicted interactions) in the network we followed previous learning methods that utilized a bootstrap strategy [19,20,42]. For each iteration of the bootstrap we first randomly sample 80% of all single cells in the dataset. Next, we used these cells to determine cell type frequencies in each sample and to perform the discretization and network learning as described above. This step is repeated 100 times, and for which we counted the presence of all directed edges. While the direction of an edge in a Bayesian network does not always imply casual interactions [43], we observed that high scoring differential edges were also very consistent in their direction (**S1 Table**).

### Ligand-Target Regression (LTR) model

The bootstrapping method presented above provides a small set of high scoring differential interactions between some of the cell types in the dataset. To obtain a mechanistic explanation for these interactions, and to identify the interacting genes between the two cell types we focused on ligand-target interactions between the two cells types. Specifically, for a predicted directed edge between cell types A and B we learned a Ligand-Target Regression (LTR) model to determine if there is an underlying cell type–cell type interaction between A and B. Our assumption is that if these two cell types indeed interact, then the expression of some of the ligands in cell A type should be able to explain some of the expression changes observed in cell type B. Similar approaches have been used by others to explore cell-type interactions in non case control studies [7].To identify a set of ligands in A predicted to activate or repress target genes in B we optimized the following regression model:

$$\underset{\alpha }{\min}\;\sum_{t}^{T}{\left(\sum_{l}^{L}I_{\left(t,l\right)}L_{\left(l\right)}\alpha_{\left(l\right)}-T_{\left(t\right)}\right)}^{2}+\lambda \|\alpha \|$$ (4)

Where *I* represents an input (known) ligand-target interaction matrix [6], *L* is an input vector of log values for the expression of ligands in cell type A, *α* represents the (unobserved) ligand activation vector, *T* represents the expression levels for target genes in cell type B and *λ* is a regularization parameter. Here we used a *L1* regularization which usually leads to the selection of relatively few non zero values (corresponding to relatively few activated ligands in cell type A).

Using the inputs to sett *A*_(*t*,*l*)_ = *I*_(*t*,*l*)_*L*_(*l*)_, transforms the optimization problem to

$$\underset{\alpha }{\min}\;\sum_{t}^{T}{\left(\sum_{l}^{L}A_{\left(t,l\right)}\alpha_{\left(l\right)}-T_{\left(t\right)}\right)}^{2}+\lambda \|\alpha \|$$ (5)

Which is a standard least absolute shrinkage and selection operator (LASSO) model. To learn parameters for the model we used the “LASSOCV” function from the Python library “scikit-learn”, which implements the LASSO cross validation. Note that the model in Eq 5 using the same ligand activity parameters for all genes (there is only 1 ligand activity parameter in the model for each ligand across all target genes). Thus, we can use this model in a cross validation setting to predict the expression levels of held out targets in cell type B. For these, we know the ligand-target interaction from Matrix I and the ligand expression from L allowing us to evaluate the ability of the model to generalize to unseen targets. We also use the model to test if we obtain better prediction accuracy for significant pairs identified in the BNs.

### Training and Test for Ligand-Target Regression (LTR) model

We used a five-fold cross validation strategy to train and test the LTR model: We split the training part of each validation set into two sets to select the hyperparameter *λ* (our penalty term) and then retrain using all training data for this set and the selected *λ* to obtain the model used for the fold test data. Evaluation of predicted values is based on the average Pearson correlation between the predicted and actual expression changes for each fold. Following testing we use the average product between the log fold change and coefficient value *α* in the five-fold training models to rank the list of active ligands.

### Joint plots of Bayesian Network and LTR model scores for cell type pairs

To jointly plot the Bayesian network bootstrap score and the Pearson correlation regression score for each cell type pair, we first converted the edge count to log value. For the Pearson correlation we used the average correlation for the five-fold results. For both IPF lung data and lung aging data, cell pairs with edge count smaller than threshold of 20 are removed. To test the robustness of the method to the threshold selection we performed a validation study that tested different threshold values. We observed good agreement for values between the BN score and LTR model score, and eventually selected 20 since the Pearson correlation between BN and LTR model score reaches the high value when the threshold is set to 20, as shown in **S12 Table**. Note that for some of the pairs we tried to model using LASSO the learning terminated with coefficients of 0 for all ligands (this happened for all runs of the random interaction matrix as we mention in Results and to a few of the CV runs of the cell-cell and intra-cell models). In such cases these models were removed from the correlation analysis.

### Comparison to CellPhoneDB, iTALK and NicheNet

All the three methods are based on ligand related gene expression analysis. For CellPhoneDB, the result contains all possible cell type pairs with calculated ligand-receptor scores. For each cell type pair, we use the sum of all its ligand-receptor scores as its pair score. iTALK detects significant ligand- receptor pairs, and provides the mean expression level of them. We then sum the product of ligand and receptor expression as the final score for each cell type pair. NicheNet can select top functional ligand genes with prediction scores for a given cell type pair. We next sum these prediction scores for all selected ligands to rank cell type pairs.

## Supporting information

S1 Fig Binary level of cell fractions for IPF lung data using GMM with two components. Sometimes GMM would assign these with larger fraction values as “0”, like for cDC1 cell. In such case, the labels would be corrected.
(TIF)

S2 Fig The Bootstrapped Bayesian Network based on the whole lung data with control and IPF results. In the network, there are edges between the same general cell types, edges between cell types from different general cell types and edges between disease node and related cell types.
(TIF)

S3 Fig **Comparison between original LTR model results and random interaction matrix results for IPF lung data (A) and lung aging data (B).** As can be seen, random LTR model has much less Pearson correlation results than the original model, because most of them failed to learn anything.
(TIF)

S4 Fig CellPhoneDB/iTALK/Nichenet comparison with BN for both IPF and aging data. Comparison between the CellPhoneDB/iTALK/NicheNet scores and edge counts of cell type pairs identified by BN for IPF (A) and aging data (B) respectively.
(TIF)

S5 Fig Evaluation of GMM assignments with cross validation experiments. We first learn a 2 cluster GMM using all samples. Next, we perform Leave one out (LOO) cross validation and compare it with that using all samples. Specifically, we obtain an average accuracy of 0.96 for the larger (60 samples) IPF study across the different cell types (A) and an accuracy higher than 0.9 for the smaller (15 samples) aging dataset (B).
(TIF)

S1 Table The cell type pair list detected by Bootstrap Bayesian Network for IPF and controls. (XLSX)

S2 Table The cell type pair list detected by CellPhoneDB(A)/iTALK(B)/NicheNet(C) for IPF and controls. Red cell type pairs are those supported by literature in both the interaction and direction while green cell type pairs are those having top predicted reverse pairs.
(XLSX)

S3 Table Predicted top ligand genes by LTR model for the top Bayesian Network cell type pairs in S1 Table. (XLSX)

S4 Table Top predicted ligand genes involved in Fibroblast and Lymphatic cell interaction. (XLSX)

S5 Table Top predicted ligand genes involved in Treg and fibroblast cell interaction. (XLSX)

S6 Table Top differential cell type interaction between young and old mice lung NC data by BN. (XLSX)

S7 Table Top differential cell type interaction between young and old mice lung NC data by CellPhoneDB(A)/iTALK(B)/NicheNet(C). Red cell type pairs are those supported by literature.
(XLSX)

S8 Table Top predicted differential cell type interaction enhanced by our mouse aging data. (XLSX)

S9 Table Top predicted ligand genes for cell type pairs in S8 Table. The predicted gene is labeled red if it is supported by previous studies.
(XLSX)

S10 Table The overlap cell types between ours and NC mouse aging data. The labeled cell types by different colors are merged as one cell type, respectively.
(XLSX)

S11 Table All the enhanced pairs by our own mouse aging scRNA-seq data. Red means that compared to the original bootstrap BN based on NC paper with 34 cell types in section of “Application to lung aging dataset”, these overlap cell type pairs have the same difference sign between young and old mice, while green means these overlap cell type pairs have opposite difference sign. All other cell types are those not found by the original bootstrap BN based on NC paper with 34 cell types in section of “Application to lung aging dataset”.
(XLSX)

S12 Table The model consistence (Person correlation) between BN and LTR/CellPhoneDB model of different edge count threshold for IPF lung and lung aging NC data. We selected edge count 20 as threshold finally used for both data. In each table, the left column corresponds to LTR model using cell 1 ligand to predict cell 2 target gene, the middle column corresponds to a control LTR model using cell 2 ligand to predict cell 2 target gene, and the right column corresponds to CellPhoneDB model.
(XLSX)

S13 Table Significance of permutation analysis of different edge score threshold values in aging lung data using BN and CellPhoneDB. The two methods generated 68 and 441 edges respectively. We present the percentage of selected edges of the different score thresholds for each method and the resulting p-value of the overlap.
(XLSX)

S14 Table Pearson correlation between original (80%) edge count and new percentage (60/70/90%) edge count for IPF data. (XLSX)

S15 Table Detected edges (cell type pairs) for the bootstrap analysis of Bayesian Networks when comparing IPF and healthy data using a simple discretization strategy that does not assume Gaussian distribution and is appropriate for constrained values like percentages. For each cell type, we split the values in half [min, (max+min)/2] and [(max+min)/2, max]. We next analyzed the significance of edge overlap between the two discretization strategies, and found that among 24 edges detected by the new BN, 8 are covered by the original BN result that contains 42 edges, with a p value of 7.39*10^-7 using fisher’s exact test. Such result indicates that different discretization can impact the result, but does not make a huge difference. It is noticed that all edges have the same absolute value of difference, 100, based on which the Pearson correlation between BN and LTR model would be always 0. That is to say, with such strategy, no randomization can be introduced, and the following LTR model comparison way cannot be used to validate the two models mutually.
(XLSX)

S16 Table Model agreement (Person correlation) between LTR model and BNs using the “leave one sample out” (LOSO) strategy that each time we leave one IPF or healthy sample out for the Bootstrapping. (XLSX)

S17 Table Model agreement between BN and LTR model using MSE (instead of Pearson correlation) as its evaluation score, for different edge count threshold on IPF lung data. LTR control model represents the model that cell type B’s ligand genes are used to predict B’s target genes for the BN identified edge (A->B).
(XLSX)

S18 Table BN Performance on simulation data with different drop-out rate. The true positive ones are red and false positive ones are blue.
(XLSX)

S19 Table Quantification of BN performance on simulation data. Here Accuracy = #true positive /# ground truth, and Precision = #true positive /# predicted as positive.
(XLSX)

S1 Text Single-cell barcoding, library preparation, and sequencing of Fendrr-floxed Mice. (DOCX)

S2 Text Fastq generation, identification of valid cell barcodes and generation of the gene-cell-matrix from Fendrr-floxed mice. (DOCX)

S3 Text Single cell raw data preprocessing and cell type annotation. (DOCX)

S4 Text Simulated data generation. (DOCX)

## References

[1] Deng Y, Bao F, Dai Q, Wu LF, Altschuler SJ. Scalable analysis of cell-type composition from single-cell transcriptomics using deep recurrent learning. Nat Methods. 2019;16(4):311–4. Epub 2019/03/20. doi: 10.1038/s41592-019-0353-7 ; PubMed Central PMCID: PMC6774994. 30886411 PMC6774994

[2] Alavi A, Ruffalo M, Parvangada A, Huang Z, Bar-Joseph Z. A web server for comparative analysis of single-cell RNA-seq data. Nat Commun. 2018;9(1):4768. Epub 2018/11/15. doi: 10.1038/s41467-018-07165-2 ; PubMed Central PMCID: PMC6233170. 30425249 PMC6233170

[3] Kumar MP, Du J, Lagoudas G, Jiao Y, Sawyer A, Drummond DC, et al. Analysis of Single-Cell RNA-Seq Identifies Cell-Cell Communication Associated with Tumor Characteristics. Cell Rep. 2018;25(6):1458–68 e4. Epub 2018/11/08. doi: 10.1016/j.celrep.2018.10.047 ; PubMed Central PMCID: PMC7009724. 30404002 PMC7009724

[4] Han X, Chen H, Huang D, Chen H, Fei L, Cheng C, et al. Mapping human pluripotent stem cell differentiation pathways using high throughput single-cell RNA-sequencing. Genome Biol. 2018;19(1):47. Epub 2018/04/07. doi: 10.1186/s13059-018-1426-0 ; PubMed Central PMCID: PMC5887227. 29622030 PMC5887227

[5] Moncada R, Barkley D, Wagner F, Chiodin M, Devlin JC, Baron M, et al. Integrating microarray-based spatial transcriptomics and single-cell RNA-seq reveals tissue architecture in pancreatic ductal adenocarcinomas. Nat Biotechnol. 2020;38(3):333–42. Epub 2020/01/15. doi: 10.1038/s41587-019-0392-8 . 31932730

[6] Browaeys R, Saelens W, Saeys Y. NicheNet: modeling intercellular communication by linking ligands to target genes. Nature Methods. 2019:1–4. 31819264 10.1038/s41592-019-0667-5

[7] Efremova M, Vento-Tormo M, Teichmann SA, Vento-Tormo R. CellPhoneDB: inferring cell-cell communication from combined expression of multi-subunit ligand-receptor complexes. Nat Protoc. 2020;15(4):1484–506. Epub 2020/02/28. doi: 10.1038/s41596-020-0292-x . 32103204

[8] Adams TS, Schupp JC, Poli S, Ayaub EA, Neumark N, Ahangari F, et al. Single-cell RNA-seq reveals ectopic and aberrant lung-resident cell populations in idiopathic pulmonary fibrosis. Sci Adv. 2020;6(28):eaba1983. Epub 2020/08/25. doi: 10.1126/sciadv.aba1983 ; PubMed Central PMCID: PMC7439502. 32832599 PMC7439502

[9] Angelidis I, Simon LM, Fernandez IE, Strunz M, Mayr CH, Greiffo FR, et al. An atlas of the aging lung mapped by single cell transcriptomics and deep tissue proteomics. Nature communications. 2019;10(1):1–17. 10.1038/s41467-019-08831-9 PMC6393476 30814501

[10] Hines PJ. Stabilizing cell-type ratios. Science. 2019;366(6470):1210–. doi: 10.1126/science.366.6470.1210-c

[11] Willett RT, Bayin NS, Lee AS, Krishnamurthy A, Wojcinski A, Lao Z, et al. Cerebellar nuclei excitatory neurons regulate developmental scaling of presynaptic Purkinje cell number and organ growth. Elife. 2019;8:e50617. doi: 10.7554/eLife.50617 31742552 PMC6890462

[12] Codeluppi S, Borm LE, Zeisel A, La Manno G, van Lunteren JA, Svensson CI, et al. Spatial organization of the somatosensory cortex revealed by osmFISH. Nature methods. 2018;15(11):932–5. doi: 10.1038/s41592-018-0175-z 30377364

[13] Lee JH, Daugharthy ER, Scheiman J, Kalhor R, Yang JL, Ferrante TC, et al. Highly multiplexed subcellular RNA sequencing in situ. Science. 2014;343(6177):1360–3. doi: 10.1126/science.1250212 24578530 PMC4140943

[14] Moffitt JR, Bambah-Mukku D, Eichhorn SW, Vaughn E, Shekhar K, Perez JD, et al. Molecular, spatial, and functional single-cell profiling of the hypothalamic preoptic region. Science. 2018;362(6416):eaau5324. doi: 10.1126/science.aau5324 30385464 PMC6482113

[15] Ståhl PL, Salmén F, Vickovic S, Lundmark A, Navarro JF, Magnusson J, et al. Visualization and analysis of gene expression in tissue sections by spatial transcriptomics. Science. 2016;353(6294):78–82. doi: 10.1126/science.aaf2403 27365449

[16] Eng C-HL, Lawson M, Zhu Q, Dries R, Koulena N, Takei Y, et al. Transcriptome-scale super-resolved imaging in tissues by RNA seqFISH+. Nature. 2019;568(7751):235. doi: 10.1038/s41586-019-1049-y 30911168 PMC6544023

[17] Xia C, Fan J, Emanuel G, Hao J, Zhuang X. Spatial transcriptome profiling by MERFISH reveals subcellular RNA compartmentalization and cell cycle-dependent gene expression. Proceedings of the National Academy of Sciences. 2019;116(39):19490–9. doi: 10.1073/pnas.1912459116 31501331 PMC6765259

[18] Yuan Y, Bar-Joseph Z. GCNG: graph convolutional networks for inferring gene interaction from spatial transcriptomics data. Genome Biol. 2020;21(1):300. Epub 2020/12/12. doi: 10.1186/s13059-020-02214-w ; PubMed Central PMCID: PMC7726911. 33303016 PMC7726911

[19] Lugo-Martinez J, Ruiz-Perez D, Narasimhan G, Bar-Joseph Z. Dynamic interaction network inference from longitudinal microbiome data. Microbiome. 2019;7(1):54. Epub 2019/04/04. doi: 10.1186/s40168-019-0660-3 ; PubMed Central PMCID: PMC6446388. 30940197 PMC6446388

[20] Imoto S, Kim SY, Shimodaira H, Aburatani S, Tashiro K, Kuhara S, et al. Bootstrap analysis of gene networks based on Bayesian networks and nonparametric regression. Genome Informatics. 2002;13:369–70.

[21] Kia’i N, Bajaj T. Histology, Respiratory Epithelium. StatPearls. Treasure Island (FL) 2020. 31082105

[22] Qiu H, He Y, Ouyang F, Jiang P, Guo S, Guo Y. The Role of Regulatory T Cells in Pulmonary Arterial Hypertension. J Am Heart Assoc. 2019;8(23):e014201. Epub 2019/11/28. doi: 10.1161/JAHA.119.014201 ; PubMed Central PMCID: PMC6912965. 31771439 PMC6912965

[23] Noubade R, Majri-Morrison S, Tarbell KV. Beyond cDC1: Emerging Roles of DC Crosstalk in Cancer Immunity. Front Immunol. 2019;10:1014. Epub 2019/05/31. doi: 10.3389/fimmu.2019.01014 ; PubMed Central PMCID: PMC6521804. 31143179 PMC6521804

[24] Wang Y, Wang R, Zhang S, Song S, Jiang C, Han G, et al. iTALK: an R Package to Characterize and Illustrate Intercellular Communication. bioRxiv. 2019:507871. doi: 10.1101/507871

[25] Dickey BF, Whitsett JA. Understanding Interstitial Lung Disease: It’s in the Mucus. Am J Respir Cell Mol Biol. 2017;57(1):12–4. Epub 2017/07/01. doi: 10.1165/rcmb.2017-0116ED ; PubMed Central PMCID: PMC5516287. 28665223 PMC5516287

[26] Platonova N, Miquel G, Regenfuss B, Taouji S, Cursiefen C, Chevet E, et al. Evidence for the interaction of fibroblast growth factor-2 with the lymphatic endothelial cell marker LYVE-1. Blood, The Journal of the American Society of Hematology. 2013;121(7):1229–37. doi: 10.1182/blood-2012-08-450502 23264596

[27] Avraham T, Daluvoy S, Zampell J, Yan A, Haviv YS, Rockson SG, et al. Blockade of transforming growth factor-beta1 accelerates lymphatic regeneration during wound repair. Am J Pathol. 2010;177(6):3202–14. Epub 2010/11/09. doi: 10.2353/ajpath.2010.100594 ; PubMed Central PMCID: PMC2993295. 21056998 PMC2993295

[28] Doucet C, Brouty-Boye D, Pottin-Clemenceau C, Canonica GW, Jasmin C, Azzarone B. Interleukin (IL) 4 and IL-13 act on human lung fibroblasts. Implication in asthma. J Clin Invest. 1998;101(10):2129–39. Epub 1998/05/29. doi: 10.1172/JCI741 ; PubMed Central PMCID: PMC508801. 9593769 PMC508801

[29] Coker RK, Laurent GJ, Shahzeidi S, Lympany PA, du Bois RM, Jeffery PK, et al. Transforming growth factors-beta 1, -beta 2, and -beta 3 stimulate fibroblast procollagen production in vitro but are differentially expressed during bleomycin-induced lung fibrosis. Am J Pathol. 1997;150(3):981–91. Epub 1997/03/01. ; PubMed Central PMCID: PMC1857875. 9060836 PMC1857875

[30] Kara Rogers Senior Editor BS. The Respiratory System: Britannica Educational Pub.; 2010.

[31] Bergamaschi E, Canu IG, Prina-Mello A, Magrini A. Biomonitoring. Adverse effects of engineered nanomaterials: Elsevier; 2017. p. 125–58.

[32] Acton QA. Asthma: New Insights for the Healthcare Professional: 2013 Edition: ScholarlyEditions; 2013.

[33] Sabbione F, Gabelloni ML, Ernst G, Gori MS, Salamone G, Oleastro M, et al. Neutrophils suppress gammadelta T-cell function. Eur J Immunol. 2014;44(3):819–30. Epub 2013/11/26. doi: 10.1002/eji.201343664 . 24271816

[34] Rodriguez-Pinto D, Saravia NG, McMahon-Pratt D. CD4 T cell activation by B cells in human Leishmania (Viannia) infection. BMC Infect Dis. 2014;14:108. Epub 2014/02/27. doi: 10.1186/1471-2334-14-108 ; PubMed Central PMCID: PMC3937821. 24568275 PMC3937821

[35] Salam N, Rane S, Das R, Faulkner M, Gund R, Kandpal U, et al. T cell ageing: effects of age on development, survival & function. Indian J Med Res. 2013;138(5):595–608. Epub 2014/01/18. ; PubMed Central PMCID: PMC3928693. 24434315 PMC3928693

[36] Grivennikov SI, Tumanov AV, Liepinsh DJ, Kruglov AA, Marakusha BI, Shakhov AN, et al. Distinct and nonredundant in vivo functions of TNF produced by t cells and macrophages/neutrophils: protective and deleterious effects. Immunity. 2005;22(1):93–104. Epub 2005/01/25. doi: 10.1016/j.immuni.2004.11.016 . 15664162

[37] Lahn M, Kalataradi H, Mittelstadt P, Pflum E, Vollmer M, Cady C, et al. Early preferential stimulation of gamma delta T cells by TNF-alpha. J Immunol. 1998;160(11):5221–30. Epub 1998/05/30. . 9605117

[38] TNFSF18 TNF superfamily member 18 [Homo sapiens (human)] 2020 [updated 22-Aug-2020]. Available from: https://www.ncbi.nlm.nih.gov/gene/8995.

[39] Yang H, Wang H, Jaenisch R. Generating genetically modified mice using CRISPR/Cas-mediated genome engineering. Nat Protoc. 2014;9(8):1956–68. Epub 2014/07/25. doi: 10.1038/nprot.2014.134 . 25058643

[40] Stuart T, Butler A, Hoffman P, Hafemeister C, Papalexi E, Mauck WM 3rd, et al. Comprehensive Integration of Single-Cell Data. Cell. 2019;177(7):1888–902 e21. Epub 2019/06/11. doi: 10.1016/j.cell.2019.05.031 ; PubMed Central PMCID: PMC6687398. 31178118 PMC6687398

[41] Aliferis CF, Statnikov A, Tsamardinos I, Mani S, Koutsoukos XD. Local causal and markov blanket induction for causal discovery and feature selection for classification part i: Algorithms and empirical evaluation. Journal of Machine Learning Research. 2010;11(Jan):171–234.

[42] Friedman N, Goldszmidt M, Wyner AJ, editors. Data Analysis with Bayesian Networks: A Bootstrap Approach. UAI; 1999.

[43] Nadkarni S, Shenoy PP. A Bayesian network approach to making inferences in causal maps. European Journal of Operational Research. 2001;128(3):479–98.

[44] Chua RL, Lukassen S, Trump S, Hennig BP, Wendisch D, Pott F, et al. COVID-19 severity correlates with airway epithelium-immune cell interactions identified by single-cell analysis. Nat Biotechnol. 2020;38(8):970–9. Epub 2020/06/28. doi: 10.1038/s41587-020-0602-4 . 32591762

[45] Morgado FN, da Silva AVA, Porrozzi R. Infectious Diseases and the Lymphoid Extracellular Matrix Remodeling: A Focus on Conduit System. Cells. 2020;9(3). Epub 2020/03/20. doi: 10.3390/cells9030725 ; PubMed Central PMCID: PMC7140664. 32187985 PMC7140664

[46] Kumagai Y, Takeuchi O, Kato H, Kumar H, Matsui K, Morii E, et al. Alveolar macrophages are the primary interferon-alpha producer in pulmonary infection with RNA viruses. Immunity. 2007;27(2):240–52. Epub 2007/08/29. doi: 10.1016/j.immuni.2007.07.013 . 17723216

[47] Chaudhuri V, Karasek MA. Mechanisms of microvascular wound repair II. Injury induces transformation of endothelial cells into myofibroblasts and the synthesis of matrix proteins. In Vitro Cell Dev Biol Anim. 2006;42(10):314–9. Epub 2007/02/24. doi: 10.1290/0607044.1 . 17316065

[48] Gochhait D, Dey P, Verma N. Cytology of plasma cell rich effusion in cases of plasma cell neoplasm. J Cytol. 2016;33(3):150–3. Epub 2016/10/21. doi: 10.4103/0970-9371.177147 ; PubMed Central PMCID: PMC4995873. 27756988 PMC4995873

[49] Smyth LCD, Rustenhoven J, Scotter EL, Schweder P, Faull RLM, Park TIH, et al. Markers for human brain pericytes and smooth muscle cells. J Chem Neuroanat. 2018;92:48–60. Epub 2018/06/11. doi: 10.1016/j.jchemneu.2018.06.001 . 29885791

[50] Sweeney M, Foldes G. It Takes Two: Endothelial-Perivascular Cell Cross-Talk in Vascular Development and Disease. Front Cardiovasc Med. 2018;5:154. Epub 2018/11/15. doi: 10.3389/fcvm.2018.00154 ; PubMed Central PMCID: PMC6218412. 30425990 PMC6218412

