# Supplementary figures and images for: CINS: Cell Interaction Network inference from Single cell expression data
Source: PLoS Comput Biol. 2022 Sep 12;18(9):e1010468. doi: 10.1371/journal.pcbi.1010468 (PMC9499239; doi:10.1371/journal.pcbi.1010468)

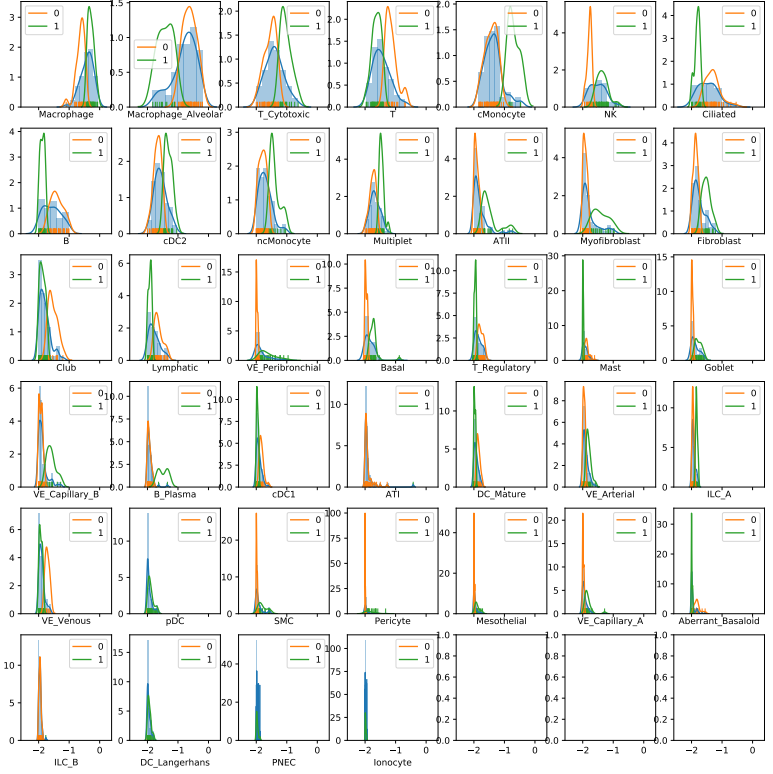

Supplement: S1 Fig — Sometimes GMM would assign these with larger fraction values as “0”, like for cDC1 cell. In such case, the labels would be corrected. (TIF) [file pcbi.1010468.s001.tif]

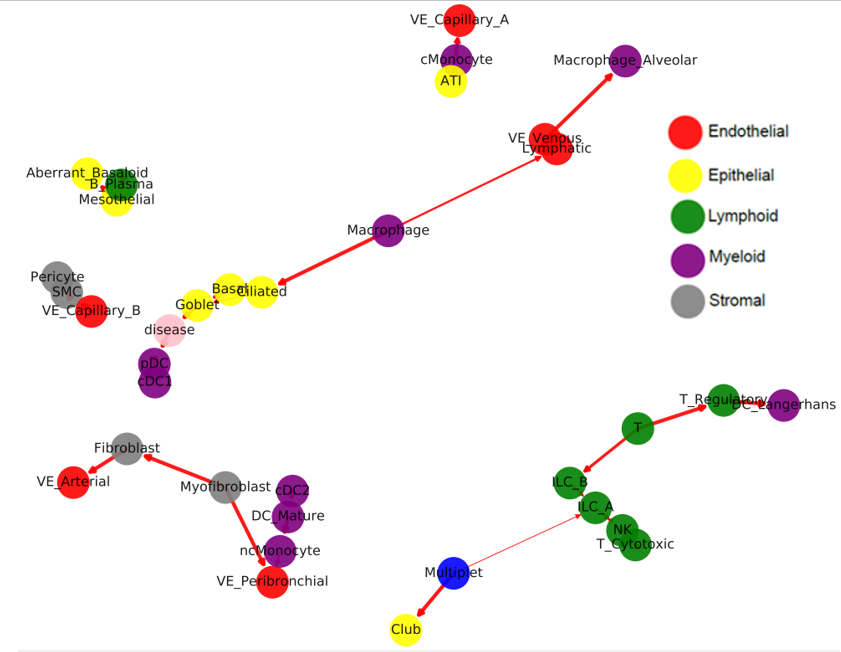

Supplement: S2 Fig — In the network, there are edges between the same general cell types, edges between cell types from different general cell types and edges between disease node and related cell types. (TIF) [file pcbi.1010468.s002.tif]

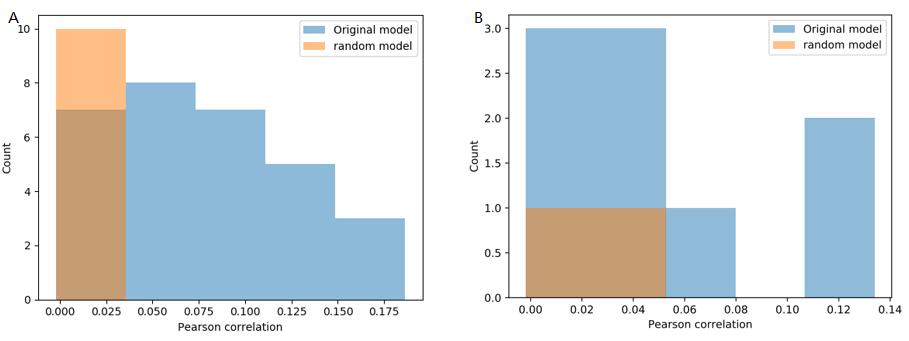

Supplement: S3 Fig — Comparison between original LTR model results and random interaction matrix results for IPF lung data (A) and lung aging data (B). As can be seen, random LTR model has much less Pearson correlation results than the original model, because most of them failed to learn anything. (TIF) [file pcbi.1010468.s003.tif]

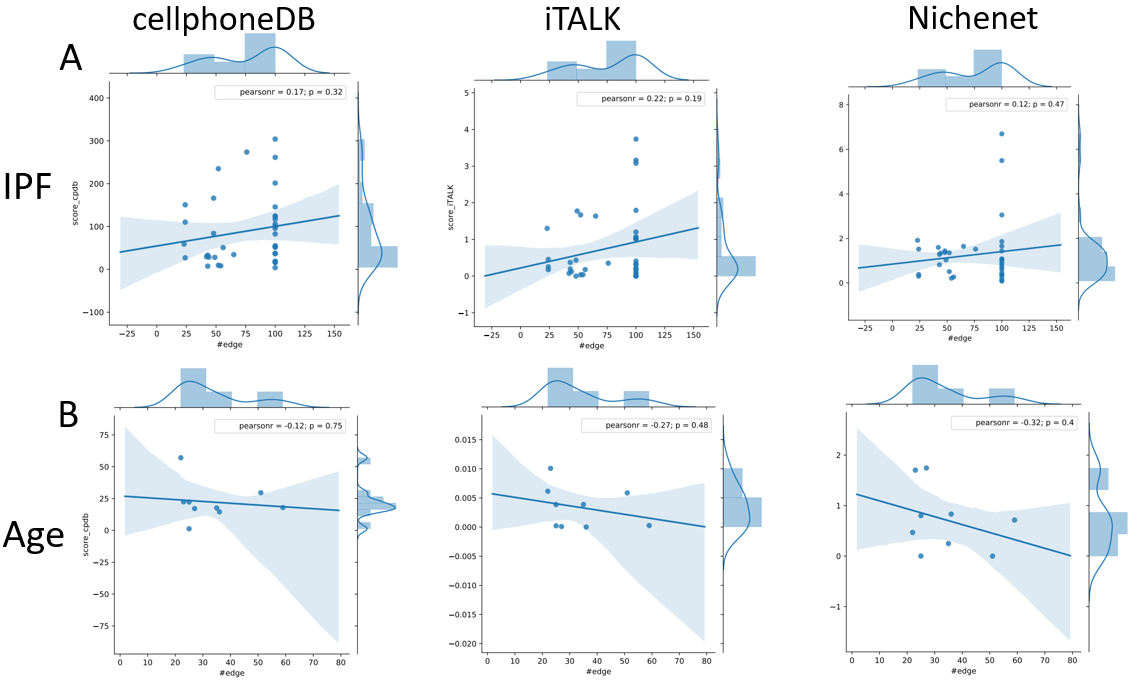

Supplement: S4 Fig — Comparison between the CellPhoneDB/iTALK/NicheNet scores and edge counts of cell type pairs identified by BN for IPF (A) and aging data (B) respectively. (TIF) [file pcbi.1010468.s004.tif]

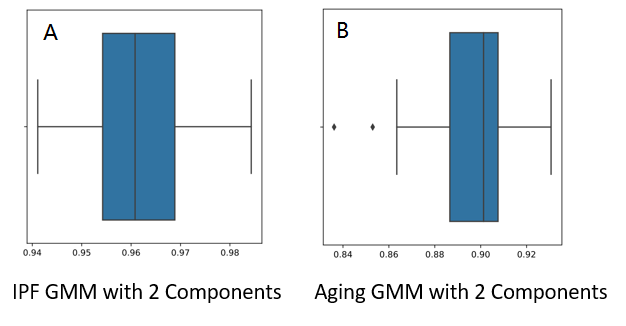

Supplement: S5 Fig — We first learn a 2 cluster GMM using all samples. Next, we perform Leave one out (LOO) cross validation and compare it with that using all samples. Specifically, we obtain an average accuracy of 0.96 for the larger (60 samples) IPF study across the different cell types (A) and an accuracy higher than 0.9 for the smaller (15 samples) aging dataset (B). (TIF) [file pcbi.1010468.s005.tif]
