# Supplementary material for: CINS: Cell Interaction Network inference from Single cell expression data
Source: PLoS Comput Biol. 2022 Sep 12;18(9):e1010468. doi: 10.1371/journal.pcbi.1010468 (PMC9499239; doi:10.1371/journal.pcbi.1010468)
Supplement: S1 Text — (DOCX) [file pcbi.1010468.s025.docx]

**S1 Text. Single-cell barcoding, library preparation, and sequencing of *Fendrr-floxed* Mice**

Briefly, single cell suspensions, reverse transcription master mix, and partitioning oil were loaded onto a “A” chip with a targeted cell output of 10,000 cells per library and then run on the Chromium Controller. After reverse transcription, cDNA was amplified for 12 cycles total. cDNA was fragmented using the proprietary fragmentation enzyme, followed by end-repair and A-tailing. Sequencing adaptors were ligated to the cDNA, then cDNA was amplified using a sample-specific index oligo as a primer. Clean-up and size selection steps were using SPRIselect beads. cDNA and final libraries were analyzed on an Agilent Bioanalyzer High Sensitivity DNA chip for qualitative control purposes. Final cDNA libraries were sequenced on an Illumina HiSeq 4000 with a sequencing configuration of 26 base pair (bp) on read1 and 98 bp on read2 aiming for 150 million reads per library.
