# Supplementary material for: CINS: Cell Interaction Network inference from Single cell expression data
Source: PLoS Comput Biol. 2022 Sep 12;18(9):e1010468. doi: 10.1371/journal.pcbi.1010468 (PMC9499239; doi:10.1371/journal.pcbi.1010468)
Supplement: S2 Text — (DOCX) [file pcbi.1010468.s026.docx]

**S2 Text. Fastq generation, identification of valid cell barcodes and generation of the gene-cell-matrix from *Fendrr-floxed* mice**

Basecalls were converted to reads using 10x Genomics CellRanger’s (v2.2) implementation of mkfastq. Contaminants on Read2 were trimmed with cutadapt (v1.17) for a) the template switch oligo sequence (AAGCAGTGGTATCAACGCAGAGTACATGGG) anchored on the 5′ end and b) poly(A) sequences on the 3′ end. Read pairs were discarded if the trimmed read2 was below 25 bp. Reads were processed using the zUMIs pipeline (v2.4.5). Paired reads were discarded if either the cell barcode or unique molecular identifier (UMI) sequence had more than 1 bp with a phred of <20. Reads were aligned to the ensemble mouse reference genome GRCm38 release 96 using the mapping software STAR (v2.6.0c). Collapsed UMIs originating from RNA molecules that span both exonic and intronic sequences were retained as both separate and combined gene expression assays. zUMI’s output of gene IDs was in ensemble format. To enable the interpretability, gene IDs were converted to Hugo Gene Nomenclature Committee (HGNC) gene names using the R package BioMart only if an exact one-to-one translation was available. High quality cell barcodes were delineated from barcodes of dying cells or background RNA based on the following thresholds: a) at least 5% of transcripts arising from nascent, unspliced mRNA; b) at least 1000 unique RNA molecules profiled; and c) less than 4% of their transcriptome was of mitochondrial origin. Generated sequencing data is available at GEO accession number GSE165638.
