# Supplementary material for: CINS: Cell Interaction Network inference from Single cell expression data
Source: PLoS Comput Biol. 2022 Sep 12;18(9):e1010468. doi: 10.1371/journal.pcbi.1010468 (PMC9499239; doi:10.1371/journal.pcbi.1010468)
Supplement: S3 Text — (DOCX) [file pcbi.1010468.s027.docx]

**S3 Text. Single cell raw data preprocessing and cell type annotation**

Principal Component Analysis (PCA) was performed using the scaled data and the 3,500 variable genes calculated determined for the dataset. Of the 100 principal components (PCs) calculated by the PCA algorithm, an assessment of an elbow plot showing the percent variance captured by each principal component (PC) revealed that the first 75 PCs were of potential interest, and they were selected for clustering and the following Uniform Manifold Approximation and Projection (UMAP). Briefly, a K-Nearest Neighbors (KNN) network was constructed through the *FindNeighbors* function with the default parameters with PCs 1 to 75 as dimensionality input. Next, the KNN network was fed into the *FindClusters* function that uses the Louvain algorithm to cluster the cells by shared patterns in gene expression. Finally, the clustered data was subjected to another round of dimensionality reduction for two-dimensional graph embedding through UMAP machine learning technique. The quality of subject and age representation within each cluster was assessed prior to cell type annotation to note any subject- or age-specific biases.
