# Supplementary material for: CINS: Cell Interaction Network inference from Single cell expression data
Source: PLoS Comput Biol. 2022 Sep 12;18(9):e1010468. doi: 10.1371/journal.pcbi.1010468 (PMC9499239; doi:10.1371/journal.pcbi.1010468)
Supplement: S4 Text — (DOCX) [file pcbi.1010468.s028.docx]

**S4 Text. Simulated data generation**

For the simulation, we assumed 10 cell types ($x_{0-9}$) each with 2,000 genes (cell types differ by the mean of expression levels of these genes). We also generated a BN for cell type interactions as specified below. Next, we used the (generative) BN model to sample cells for each type as follows:

A synthetic dataset including 10 cell types are constructed. In the BN, the first 6 cell types $(x_{0},x_{1}, x_{2}, x_{3},x_{4}\mathrm{and}x_{5})$ do not have parents. The other nodes have 1 or 2 parent nodes in the network and their CPTs are listed below:

$$P(x_{6})=N(x_{0},\sigma),$$

$$P(x_{7})=N(2*x_{1},\sigma),$$

$$P(x_{8})={N(x}_{2}+x_{3},\sigma),$$

$$P(x_{9})=N(x_{4}+2*x_{5},\sigma),$$

Where N represents Gaussian distribution and $\sigma$ is the variance associated with the Gaussian model. The total number of edges in the network is thus 6 (see also column 1 of Table R1).

We next used the BN to determine the number of cells for each type and then sampled 2,000 genes for each cell based on the distribution for that cell type. We also tested the impact of dropouts and dropouts with several settings. We repeated this process to sample 100 individuals from the BN. Finally, we used the set of generated samples to learn a BN using CINS.
